# Predicting the unpredictable: a robust nomogram for predicting recurrence in patients with ampullary carcinoma

**DOI:** 10.1186/s12885-024-11960-0

**Published:** 2024-02-15

**Authors:** Ruiqiu Chen, Lin Zhu, Yibin Zhang, Dongyu Cui, Ruixiang Chen, Hao Guo, Li Peng, Chaohui Xiao

**Affiliations:** 1 Medical School of Chinese PLA, Beijing, China; 2 Faculty of Hepato-Biliary-Pancreatic Surgery, the First Medical Centre, Chinese People s Liberation Army (PLA) General Hospital, Beijing, China; 3 The First School of Clinical Medicine, Lanzhou University, No. 1, Donggangxi Rd, Chengguan District, 730000 Lanzhou, Gansu China; 4 The Fourth Hospital of Hebei Medical University, Shijiazhuang, China; 5 Key Laboratory of Digital Hepatobiliary Surgery PLA, Beijing, China; 6 Hebei Medical University, Shijiazhuang, China; 7 Department of Hepatobiliary Surgery, Zhongshan Hospital of Xiamen University, Xiamen, Fujian China

**Keywords:** Ampullary Carcinoma, Recurrence, Lasso-Cox regression, Prediction model, Nomogram

## Abstract

**Objective:**

To screen the risk factors affecting the recurrence risk of patients with ampullary carcinoma (AC)after radical resection, and then to construct a model for risk prediction based on Lasso-Cox regression and visualize it.

**Methods:**

Clinical data were collected from 162 patients that received pancreaticoduodenectomy treatment in Hebei Provincial Cancer Hospital from January 2011 to January 2022. Lasso regression was used in the training group to screen the risk factors for recurrence. The Lasso-Cox regression and Random Survival Forest (RSF) models were compared using Delong test to determine the optimum model based on the risk factors. Finally, the selected model was validated using clinical data from the validation group.

**Results:**

The patients were split into two groups, with a 7:3 ratio for training and validation. The variables screened by Lasso regression, such as CA19-9/GGT, AJCC 8th edition TNM staging, Lymph node invasion, Differentiation, Tumor size, CA19-9, Gender, GPR, PLR, Drinking history, and Complications, were used in modeling with the Lasso-Cox regression model (C-index = 0.845) and RSF model (C-index = 0.719) in the training group. According to the Delong test we chose the Lasso-Cox regression model (*P* = 0.019) and validated its performance with time-dependent receiver operating characteristics curves(tdROC), calibration curves, and decision curve analysis (DCA). The areas under the tdROC curves for 1, 3, and 5 years were 0.855, 0.888, and 0.924 in the training group and 0.841, 0.871, and 0.901 in the validation group, respectively. The calibration curves performed well, as well as the DCA showed higher net returns and a broader range of threshold probabilities using the predictive model. A nomogram visualization is used to display the results of the selected model.

**Conclusion:**

The study established a nomogram based on the Lasso-Cox regression model for predicting recurrence in AC patients. Compared to a nomogram built via other methods, this one is more robust and accurate.

**Supplementary Information:**

The online version contains supplementary material available at 10.1186/s12885-024-11960-0.

## Introduction

Ampullary carcinoma is a rare malignant tumor that starts in the small intestine at the junction of bile and pancreatic ducts [1]. 。Its incidence accounts for 0.2 -0.5% of digestive tract malignant tumors and 6 -17% of periampullary tumors [2]. One of the primary treatment options for ampullary carcinoma (AC) is Pancreaticoduodenectomy (PD). The AC prognosis tends to be superior to that of other peripelvic tumors. The prognosis for AC, however, is not satisfactory after extensive research and follow-up, particularly because the overall survival of patients who experience a recurrence after PD is relatively poor [3]. Therefore, it is crucial to identify and promptly treat individuals at high risk of recurrence to improve the prognosis of AC patients.

To date, there are few prognostic scoring systems available for AC, even though numerous research studies have integrated demographic and clinical data to build risk-scoring systems for gastrointestinal cancers [4–6]. The American Joint Committee on Cancer (AJCC) 8th Tumor-Node-Metastasis (TNM) staging is widely used as a gold standard for evaluating patients’ disease progression and prognosis in clinical practice. However, patients with similar scores in this system can have greatly differing prognostic survival rates. On the other hand, some researchers have attempted to assess patient prognosis at multiple levels, including genes and cytokines [7, 8]. However, there are obvious limitations, including more complicated technical requirements, expensive monitoring index equipment, and difficult accessibility in the clinic. Consequently, It is essential to create a reliable and accurate prediction model for AC patients.

Many studies have shown that the nomogram is a statistical model for individualized analysis of clinical events that can quantify the risk of clinical events through multiple factors and support the prevention and treatment of clinical events [9, 10]. Most nomogram parameter screening processes are based on univariate and multivariate analyses, which have limitations in dealing with multicollinearity between variables. Less widely utilized in the field of AC, lasso regression has the benefit of allowing for the construction of more accurate and robust models through the construction of a penalty function [11, 12]. However, few studies have utilized the Lasso- cox method for modeling in terms of the nomogram of AC. Based on clinicopathologic characteristics and prognostic factors, a combination of Lasso regression and Cox regression was utilized in this investigation. The former allows for effective screening of variables, while the latter allows for modeling and visualization for direct interpretation; subsequently, a more rigorous correlation validation was performed by a correlation validation group.

The purpose of this study is to construct a novel model based on the Lasso-Cox model for predicting the recurrence of AC to further comprehend patient disease characteristics and provide customized treatment plans. Meanwhile, it will be able to identify high-risk individuals and expose them to more thorough follow-up and surveillance, allowing clinicians to catch the return or advancement of the disease early on and take appropriate action. This will be an indispensable step towards precision medicine.

## Materials and methods

### Patients enrolled

162 ampullary cancer patients who had experienced pancreaticoduodenectomy at Hebei Provincial Cancer Hospital between January 2011 and January 2022 were included in this study. This study was approved by the Medical Ethics Committee of Hebei Provincial Cancer Hospital. Inclusion criteria: (1) preoperative evaluation met the indication for radical surgical resection and radical pancreaticoduodenectomy was performed; (2) postoperative pathology diagnosed AC; patients were excluded if any of the following conditions were fulfilled: (1) The patient underwent preoperative adjuvant therapy; (2) patients died as a result of other illnesses or unexpected events; and (3) patients’ clinical and follow-up data were not available. A prediction model was established in this study based on the clinicopathological characteristics of the patients, inflammatory markers, and the tumor’s grading and staging. The included patients were then randomly split into a training group (N1 = 114) and a validation group (N2 = 48) in a ratio of 7:3, and the demographics, laboratory results, and prognosis of the patients in the two groups were compared to establish a more reliable and robust model. The clinicopathologic staging in this article was guided by the AJCC 8th edition criteria for AC [13].

### Clinicopathological data collection

Numerous demographic data, clinicopathologic characteristics, and inflammatory markers that may affect the prognosis of survival in AC were collected, including Carbohydrate antigen 19 − 9 to Gamma-glutamyltransferase Ratio (CA19-9/GGT) [14], Platelet to Lymphocyte Ratio (PLR) [15], Neutrophil to Lymphocyte Ratio (NLR) [16], Albumin to Alkaline Phosphatase Ratio (AAPR) [17], Glucose to Lymphocyte Ratio (GLR) [18], Gamma-glutamyltransferase to Platelet Ratio (GPR) [19] and Albumin to Globulin Ratio (AGR) [20]. Age, gender, drinking history, and a history of underlying illnesses were all included in the demographic information. Clinicopathological information included (1) treatment-related factors: history of preoperative jaundice reduction, blood transfusion history; (2) tumor information: tumor size, CA19-9; (3) tumor staging and grading: histological grading, AJCC 8th TNM staging system; (4) laboratory parameters: neutrophils, platelets (PLT), lymphocytes, alanine aminotransferase (ALT), total bilirubin (TBIL), albumin (ALB), Gamma-glutamyltransferase (GGT), alkaline phosphatase (ALP); (5) pathological results, postoperative complications;

Few studies have demonstrated the prognostic value of CA19-9/GGT in digestive tract malignant tumor patients [14, 21]. Therefore, CA19-9/GGT was included in this study to increase the robustness of the proposed model.

### Follow-up

The outpatient clinic, electronic contacts, and the Internet were used to keep monitor of all patients. Patients were regularly rechecked after surgery, and the rechecking items included imaging examinations and laboratory tests. The regular review was performed once a month in six months after surgery; once in three months if no signs of recurrence were seen; and once in six months for patients who had not had a recurrence for two years. Recurrence was defined as the appearance of new lesions in the vicinity of the original lesion or in other organs, and imaging reports (PET-CT, MRI) demonstrated contrast-enhanced images in certain areas. The time from diagnosis until the first recurrence or the final follow-up appointment was referred to as recurrence-free survival (RFS). Time from diagnosis to death or final follow-up was measured as overall survival (OS).

### Statistical analysis

The best cutoff values for laboratory indicators and prognostic indicators were determined using ROC curve analysis; continuous variables were expressed as mean ± standard deviation or the median ± interquartile range (IQR); categorical variables were analyzed using the chi-square test for analysis of variance; Student’s t-test and rank sum test for comparison of differences between groups. The recurrence-free survival curve was created using the Kaplan-Meier technique; Lasso regression was used to screen for the risk factors. Cox regression and random survival forest (RSF) were used for constructing two different types of prediction models using the parameters refined by Lasso regression. Delong test is used to compare the model performance. The hazard ratio (HR) value, which may be utilized as the risk score weight for assessing the prognosis, was calculated by the Cox proportional risk regression model following analysis of the prognostic variables. Using the variable importance (VIMP) approach, we determined the relative weights of each predictor in the RSF model [22]. The discrimination and consistency of the model were assessed using time-dependent ROC, C-index, and calibration curves, respectively. The clinical applicability of the method was assessed using decision curve analysis. All statistical analyses were performed using R software v4.3.1 (R Foundation for Statistical Computing, Vienna, Austria; random Forest SRC, party, party kit, and VIM packages).

## Results

### Characteristics of patients

During the study period, a total of 179 patients with ampullary tumor underwent radical pancreaticoduodenectomy, of which 2 patients were diagnosed with neuroendocrine tumors postoperatively, 3 patients died as a result of accidents, 1 patient died within 30 days of surgery, and 11 patients were excluded because of incomplete information, resulting in a total of 162 patients with AC being enrolled. Among them, 94 cases (58.02%) were male and 68 cases (41.98%) were female. Additionally, 58 cases (35.80%) of the patients had a history of drinking, 68 cases (41.98%) had underlying illnesses, 38 cases (23.46%) had preoperative jaundice reduction, and 139 cases (85.80%) had a history of perioperative blood transfusions. The gold standard for tumor clinical staging was the AJCC 8th edition staging. The “survminer” package was used to calculate optimal cutoffs for laboratory indicators, prognostic indicators, and inflammatory markers in R. Specific clinical data characteristics are detailed in the Table 1.

**Table 1:** Comparison of clinical data between training set and validation set

Variables	Total (%)	Training set (%)	Validation set (%)	p
	*n* = 162	*n* = 114	*n* = 48	
Age (years), x ± s	61.932 ± 8.390	62.140 ± 8.887	61.438 ± 7.137	0.628
Gender (Female/Male)	68 (41.98)/94 (58.02)	45 (39.47)/69 (60.53)	23 (47.92)/25 (52.08)	0.412
Drinking history (yes/no)	58 (35.80)/104 (64.20)	45 (39.47)/69 (60.53)	13 (27.08)/35 (72.92)	0.186
Underlying diseases (yes/no)	68 (41.98)/94 (58.02)	46 (40.35)/68 (59.65)	22 (45.83)/26 (54.17)	0.637
Preoperative jaundice reduction (yes/no)	38 (23.46)/124 (76.54)	26 (22.81)/88 (77.19)	12 (25.00)/36 (75.00)	0.922
Blood transfusion history (yes/no)	139 (85.80)/23 (14.20)	103 (90.35)/11 (9.65)	36 (75.00)/12 (25.00)	0.061
Complications (yes/no)	88 (54.32)/74 (45.68)	62 (54.39)/52 (45.61)	26 (54.17)/22 (45.83)	1.000
Tumor size (≤ 2.5 cm/>2.5 cm)	115 (70.99)/47 (29.01)	80 (70.18)/34 (29.82)	35 (72.92)/13 (27.08)	0.872
CA19-9/GGT (≤ 0.15/>0.15)	82 (50.62)/80 (49.38)	57 (50.00)/57 (50.00)	25 (52.08)/23 (47.92)	0.944
CA19-9 (median [IQR]) U/L	92.35 [38.91, 195.50]	92.62 [42.67, 176.06]	91.865 [27.76, 224.23]	0.689
Albumin (median [IQR]) g/L	37.95 [34.80, 41.08]	37.65 [35.23, 41.10]	38.150 [34.00, 40.40]	0.748
Alkaline phosphatase (median [IQR]) U/L	405.65 [216.43, 617.65]	405.70 [216.43, 634.93]	405.500 [227.55, 542.85]	0.834
GGT (median [IQR]) U/L	596.95 [349.15, 1008.43]	596.95 [360.23, 1016.15]	600.100 [315.35, 974.33]	0.901
TBIL (median [IQR]) µmol/L	116.75 [31.95, 252.53]	118.20 [33.95, 257.80]	116.750 [25.50, 193.63]	0.352
PLR (≤ 265.81/>265.81)	126 (77.78)/36 (22.22)	86 (75.44)/28 (24.56)	40 (83.33)/8 (16.67)	0.370
NLR (≤ 3.68/>3.68)	101 (62.35)/61 (37.65)	70 (61.40)/44 (38.60)	31 (64.58)/17 (35.42)	0.839
AAPR (≤ 0.06/>0.06)	50 (30.86)/112 (69.14)	37 (32.46)/77 (67.54)	13 (27.08)/35 (72.92)	0.624
GPR (≤ 3.75/>3.75)	120 (74.07)/42 (25.93)	86 (75.44)/28 (24.56)	34 (70.83)/14 (29.17)	0.679
GLR (≤ 385.3/>385.3)	69 (42.59)/93 (57.41)	46 (40.35)/68 (59.65)	23 (47.92)/25 (52.08)	0.474
AGR (≤ 0.08/>0.08)	102 (62.96)/60 (37.04)	71 (62.28)/43 (37.72)	31 (64.58)/17 (35.42)	0.921
Lymph node invasion (yes/no)	52 (32.10)/110 (67.90)	33 (28.95)/81 (71.05)	19 (39.58)/29 (60.42)	0.254
Perineural invasion (yes/no)	60 (37.04)/102 (62.96)	41 (35.96)/73 (64.04)	19 (39.58)/29 (60.42)	0.797
Vascular invasion (yes/no)	17 (10.49)/145 (89.51)	14 (12.28)/100 (87.72)	3 (6.25)/45 (93.75)	0.388
Diferentiation (G1/G2/G3)	11 (6.79)/116 (71.60)/35 (21.60)	8 (7.02)/80 (70.18)/26 (22.81)	3 (6.25)/36 (75.00)/9 (18.75)	0.820
AJCC 8th edition TNM stage				0.112
IA	8 (4.94)	6 (5.26)	2 (4.17)	
IB	51 (31.48)	40 (35.09)	11 (22.92)	
IIA	39 (24.07)	29 (25.44)	10 (20.83)	
IIB	12 (7.41)	6 (5.26)	6 (12.50)	
IIIA	43 (26.54)	25 (21.93)	18 (37.50)	
IIIB	9 (5.56)	8 (7.02)	1 (2.08)	

**Abbreviations: TBIL**: total bilirubin; **GGT**: gamma-glutamyltransferase; **PLR**: platelet to lymphocyte ratio; **NLR**: neutrophil to lymphocyte ratio; **AAPR**: albumin to alkaline phosphatase ratio; **GPR**: gamma-glutamyltransferase to platelet ratio; **GLR**: gamma-glutamyltransferase to lymphocyte ratio; **AGR**: albumin to gamma-glutamyltransferase ratio; **CA19-9**: carbohydrate antigen 19–9; **IQR**: Inter Quartile Range; **AJCC**: American Joint Committee on Cancer; *P*>0.05 marked in bold font shows statistical significant

The prognostic risk score was estimated by the Lasso-Cox regression model with the formula: prognostic score = (1.46 * Gender) + (1.13 * Drinking history) + (0.66 * Complications) +(0.51 * Tumor size)+ (1.17 * CA19-9) + (0.8 * CA19-9/GGT) + (0.36 * PLR) + (-0.59 * GPR) + (2.3 * Lymph node invasion) + (0.4 * Differentiation) +(0.25 * AJCC 8th edition TNM staging). (Results of the Cox proportional hazards regression were used to generate weight values)

### Prognosis-related data

The median follow-up time was 41 months (IQR = 21–59 months). 81 out of 162 individuals experienced recurrence and 73 out of 162 patients passed away after the follow-up. The 1-, 3-, and 5-year OS and RFS were 86.8%, 60.1%, 43.8%, and 68.3%, 53.6%, 40.1%, respectively. Patients were randomly assigned in a 7:3 ratio to a training group (N1 = 114) and a validation group (N2 = 48). The study’s variables included clinicopathological traits, tumor grade, stage, and pertinent inflammation-based markers. The optimal cutoff values for each prognostic indicator in the training group were calculated in R. The corresponding images were plotted in the Supplementary Figure. The optimum cutoff values for the inflammation-based markers were then utilized to plot the relevant K-M survival curves. (Supplementary Figs. 1, 2). It is necessary to develop a prognostic model for predicting recurrence to identify those who are at a high risk of recurrence and implement timely intervention. This information was obtained from the Kaplan-Meier curve between RFS and OS, which showed that OS was shorter in recurrence patients than in non-recurrence patients (Supplementary Fig. 3). The training group and the validation group performed correlation analysis, and none of the variables were statistically different between the two groups (*P* > 0.05) (Table 1). This demonstrates that randomized data grouping is logical and reasonable.

### Performance comparison between prognostic models

**Fig. 1:**
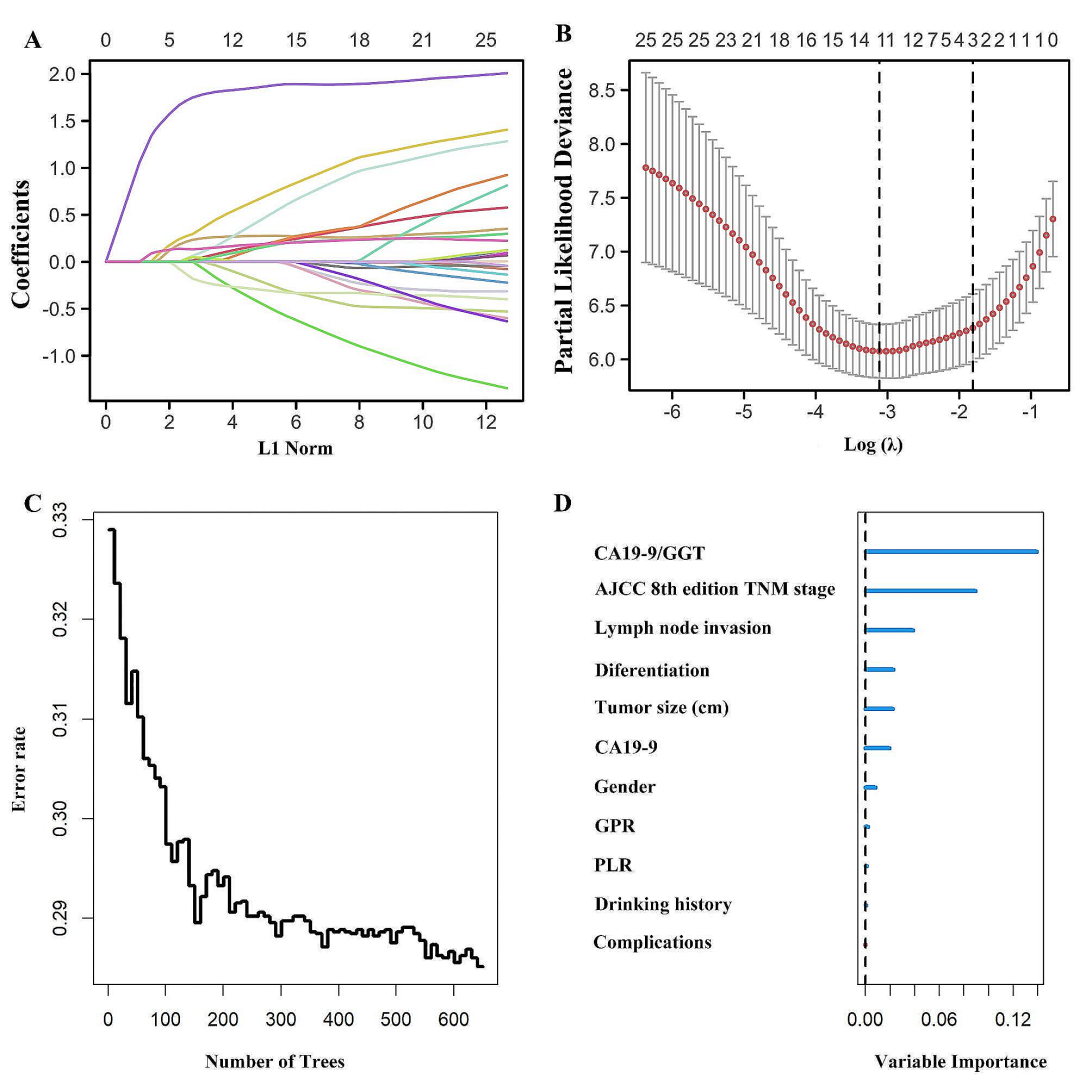
Variables screened by Lasso regression (**A**). The variation characteristics of the coefficient of variables; (**B**) The 10-fold cross-validation method is used to select the optimal value of the parameter λ in the Lasso regression model

**Fig. 2:**
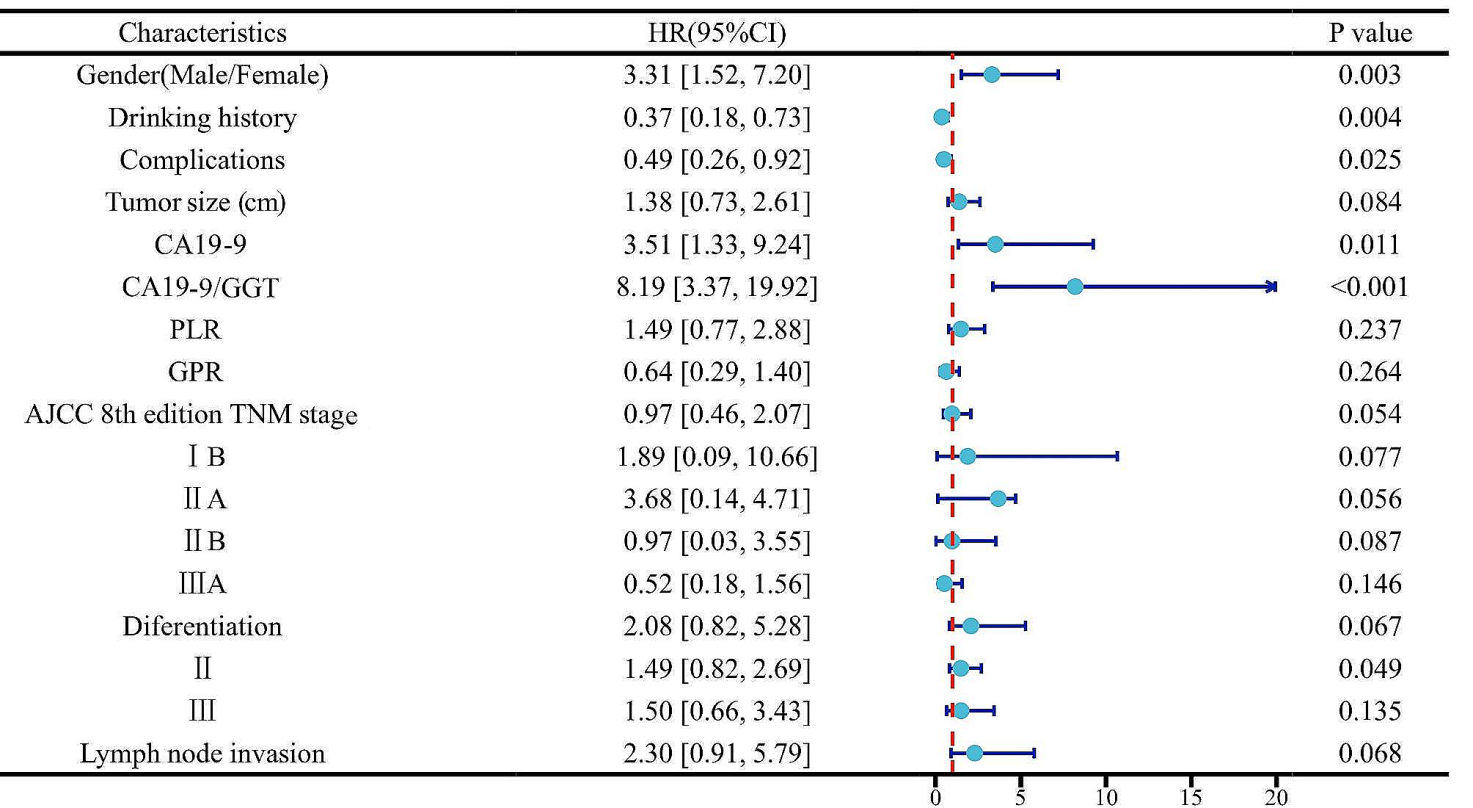
Forest plot based on Cox proportional hazards regression model in the training set

Risk factors were screened out among all variables by Lasso regression, and changes in the coefficients of each variable were characterized as shown in Fig. 1A. Then, iterative analysis was performed using 10-fold cross-validation, and a model with excellent performance was obtained when λ = 3.046 (Log λ=-1.70) (Fig. 1B). Among the screened variables were Gender, Drinking history, Complications, Tumor size, CA199, CA199/GGT, PLR, GPR, AJCC 8th edition TNM staging, differentiation, and Lymph node invasion. The Cox proportional risk regression model was further performed by the parameters screened by Lasso regression (Table 2). The C-index of the training set was calculated to be 0.845 and a forest plot was generated using Cox regression analysis (Fig. 2).

**Table 2:** Cox proportional hazards regression to predict recurrence based on Lasso regression

Variables	β	z	HR (95%CI)	*P* value
Gender (Male/Female)	1.46	3.57	3.31 [1.52, 7.20]	0.003
Drinking History	1.13	3.16	0.37 [0.18, 0.73]	0.004
Complications	0.66	2.2	0.49 [0.26, 0.92]	0.025
Tumor size (>2.5 cm/≤2.5 cm)	0.51	1.6	1.38 [0.73, 2.61]	0.084
CA19-9 (>54.87/≤54.87)	1.17	2.87	3.51 [1.33, 9.24]	0.011
CA19-9/GGT (>0.15/≤0.15)	0.80	2.11	8.19 [3.37, 19.92]	< 0.001
PLR (>265.81/≤265.81)	0.36	1.11	1.49 [0.77, 2.88]	0.237
GPR (>3.75/≤3.75)	-0.59	-1.46	0.64 [0.29, 1.40]	0.264
AJCC 8th edition TNM stage	0.25	1.94	0.97 [0.46, 2.07]	0.054
IB	0.73	0.49	1.89 [0.09, 10.66]	0.077
IIA	0.77	0.6	3.68 [0.14, 4.71]	0.056
IIB	1.58	1.16	0.97 [0.03, 3.55]	0.087
IIIA	1.78	1.31	0.52 [0.18, 1.56]	0.146
Diferentiation	0.40	1.31	2.08 [0.82, 5.28]	0.067
II	0.13	0.13	1.49 [0.82, 2.69]	0.049
III	1.02	1.01	1.50 [0.66, 3.43]	0.135
Lymph node invasion	2.30	1.42	2.30 [0.91, 5.79]	0.068

The RSF model was built in the training set utilizing the variables that were refined through Lasso regression (Fig. 1C). Through parameter debugging, the error rate of the model was stabilized when the ntree was 650, and the model’s c-index was calculated to be 0.719. According to the VIMP method, the importance of the pertinent factors was ranked in the following order: CA19-9/GGT, AJCC 8th edition TNM staging, Lymph node invasion, Differentiation, Tumor size, CA19-9, Gender, GPR, PLR, Drinking history, and Complications (Fig. 1D).

Since the C-index of the prediction model based on Lasso-Cox regression (C-index = 0.845) was higher than that of the prediction model based on random survival forest (C-index = 0.719). Meanwhile, the Lasso -Cox regression model is the optimal model (*P* = 0.019) according to the Delong test. Therefore, the prediction model established by Lasso-Cox regression was used in this study to predict the recurrence of patients with AC.

### Lasso-Cox regression model for the training group: performance and clinical applicability

**Fig. 3:**
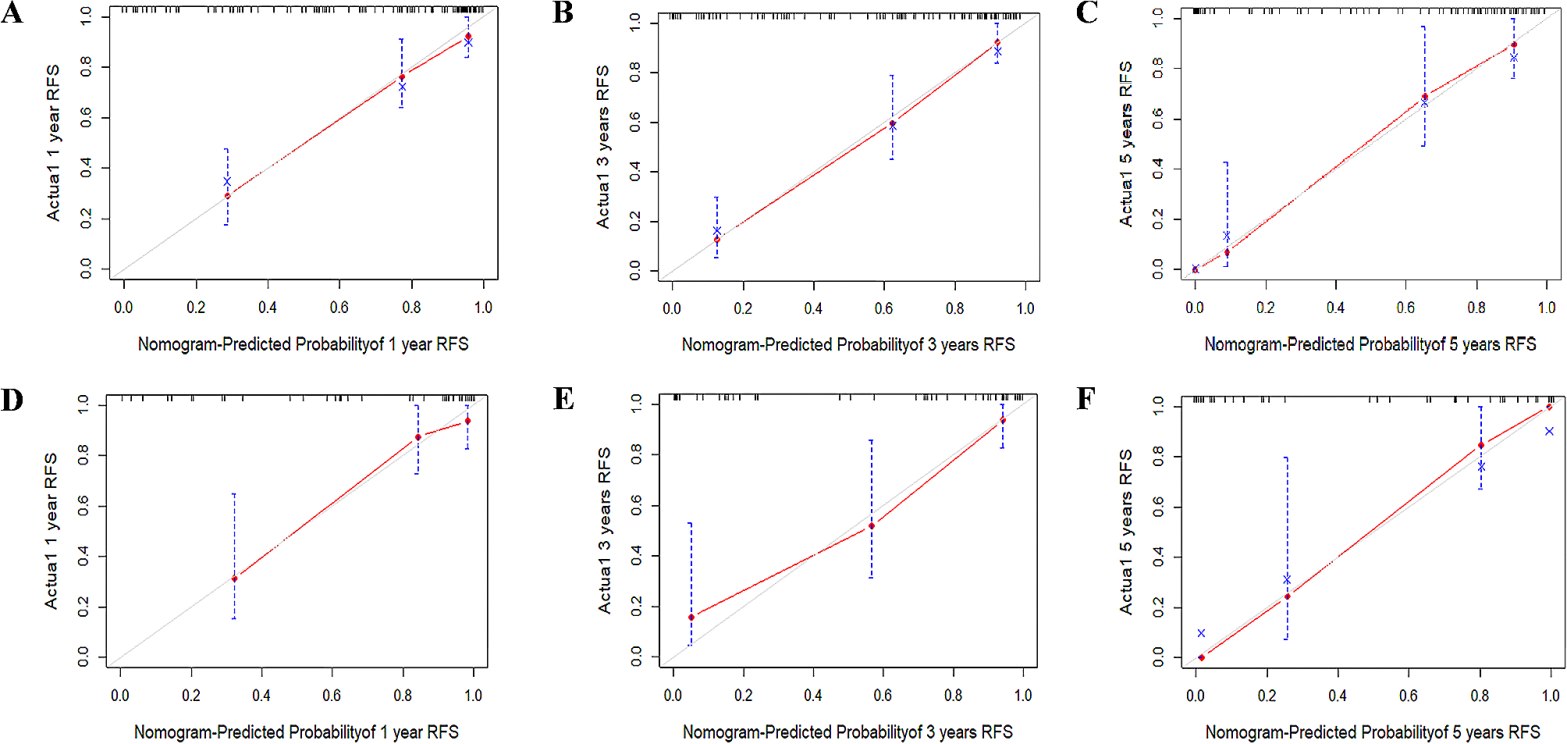
Calibration plots of predicted 1-, 3-, and 5-year RFS based on Cox regression modeling in the training set and validation set. (**A**–**C**) training set; (**D**–**F**) validation set. RFS, recurrence-free survival

**Fig. 4:**
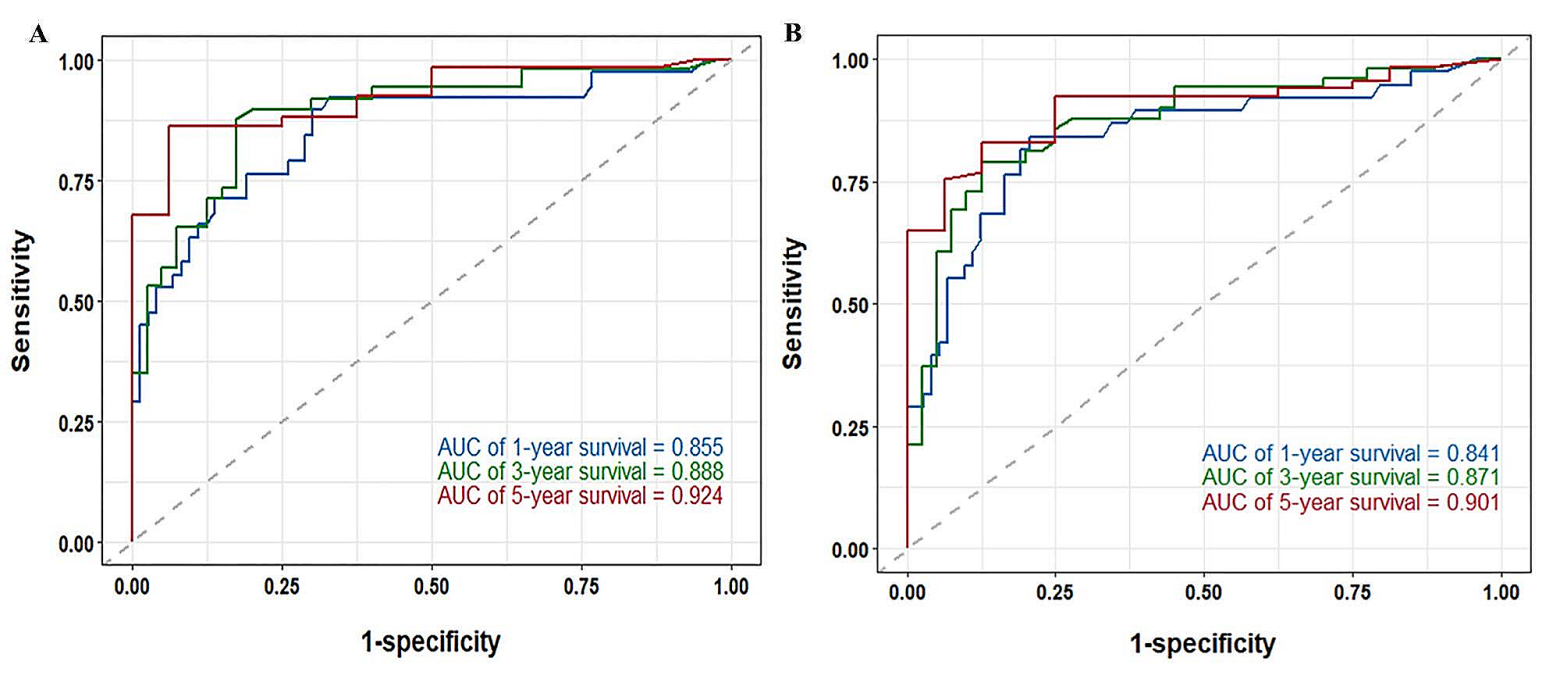
Time-dependent ROC of predicted 1-, 3-, and 5-year RFS based on Cox regression modeling in the training set and validation set. (**A**) training set; (**B**) validation set. RFS, recurrence-free survival

In comparison to the AJCC 8th TNM staging system alone (C-index = 0.746), the Lasso-Cox regression model (C-index = 0.845) demonstrated a higher C-index. The prediction model’s calibration curves for 1, 3, and 5 years demonstrated significant concordance between expected and actual results (Fig. 3A–C). At 1, 3, and 5 years, the prediction model’s area under the curve (AUC) had values of 0.855, 0.888, and 0.924, respectively (Fig. 4A). A novel assessment technique called decision curve analysis (DCA) emphasizes the net clinical benefit of prediction models [23]. Compared to the AJCC 8th TNM staging system, the nomogram produced higher net benefits over a wider range of threshold probabilities (Supplementary Fig. 4).

### Validation of the lasso-cox regression model

The validation group was brought into the optimum cut-off values of the prognostic indicators from the training group, and the K-M survival curves of the crucial variables were plotted. The results revealed that most of the optimal cutoff values for variables among the risk factors identified by Lasso regression were statistically significant (Supplementary Fig. 5) (*P* < 0.1; Note: Due to the small sample size, *P* < 0.1 was regarded as statistically significant [24, 25]). Patients from the validation group were then included in the RSF and Lasso-Cox regression models for performance comparison, respectively. Compared to the RSF model (C-index = 0.762), the C-index of the Lasso-Cox regression model (C-index = 0.867) remained higher. In comparison to the AJCC 8th TNM staging system alone, the Lasso-Cox regression model demonstrated a higher C-index (C-index = 0.758). Plotting the 1-year, 3-year, and 5-year calibration curves for the validation group revealed that the predicted and actual observations were in great concordance (Fig. 3D–F). The prediction model’s tdAUC was 0.841, 0.871, and 0.901 at 1, 3, and 5 years, respectively (Fig. 4B). Comparing the nomogram to the AJCC 8th TNM staging system in DCA, the nomogram generated larger net benefits over a wider range of threshold probabilities (Supplementary Fig. 4).

**Fig. 5:**
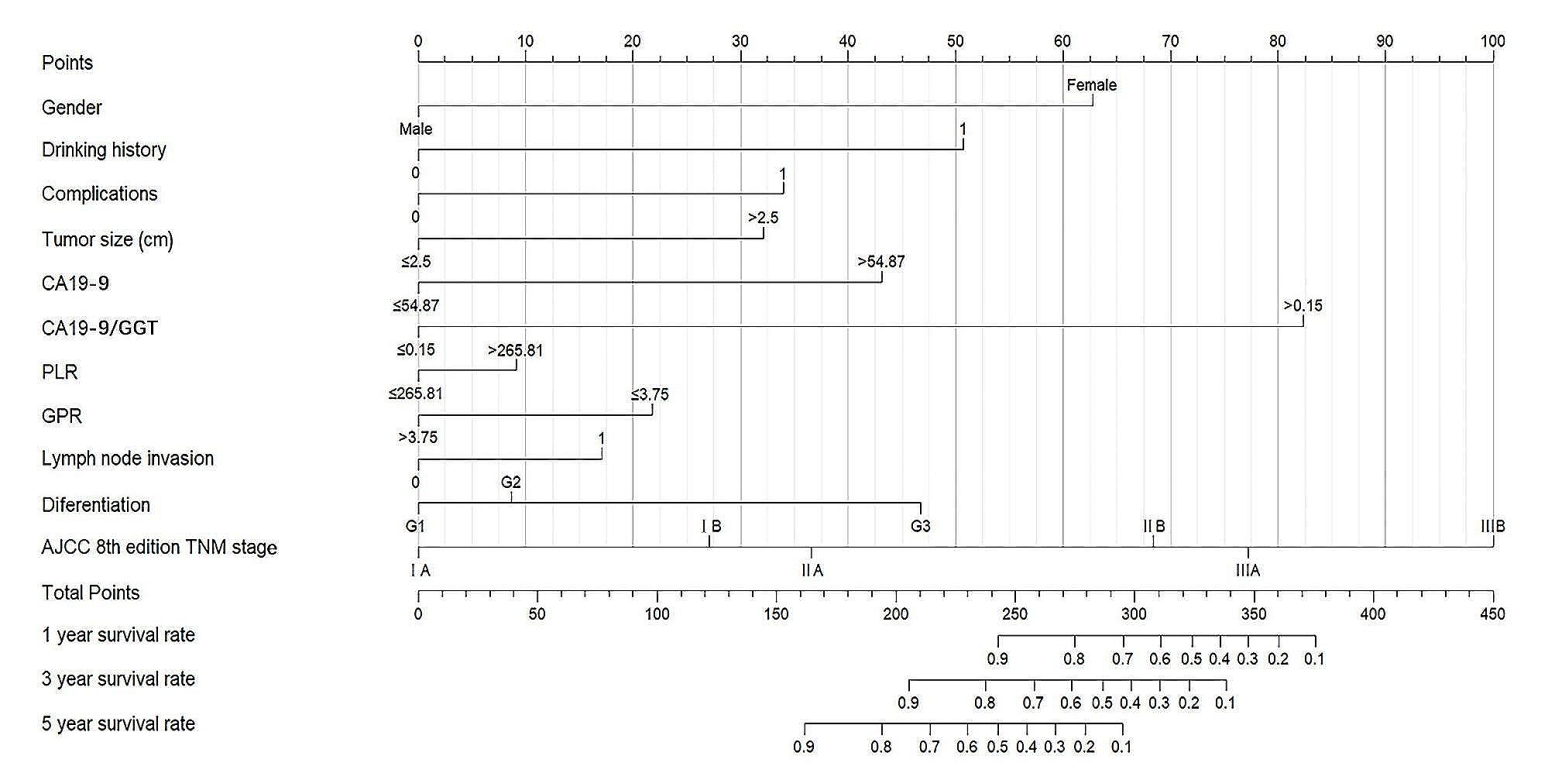
Nomogram used to predict time-related recurrence in patients with AC. AC, Ampullary carcinoma

In conclusion, the Lasso-Cox regression model was ultimately chosen to forecast the likelihood of recurrence in AC patients following radical surgery. We simplified the challenging mathematical model into a visual nomogram for straightforward clinical use (Fig. 5). The nomogram requires a summation of the scores of the variables it contains. The three lines reflecting the predicted RFS are then intersected by a vertical line at the total score. The individual’s expected 1-, 3-, and 5-year RFS rates are the numbers that correspond to the intersection points. The optimal cutoff value was calculated based on the sum of the scores for each variable in the nomogram; the accuracy of the total score was evaluated based on the Cox regression model (C-index = 0.894); and the K-M survival curve was plotted based on the optimal cutoff value (Supplementary Fig. 6) (*P* < 0.001).

## Discussion

Less research has been done on prognostic modeling for AC. To the best of our knowledge, this study is the first to incorporate inflammatory parameters and to further develop a unique predictive model using the Lasso-Cox approach. This model enables more precise and reliable identification of patients at high risk of developing recurrent AC, allowing for prompt treatment to enhance the prognosis for AC patients. Additionally, this research represents the first attempt to integrate clinicopathological characteristics, inflammatory markers, tumor stage, and grade to build a nomogram to precisely predict the probability of RFS at 1, 3, and 5 years. These clinically widespread indicators can comprehensively evaluate the specific situation of an individual, thus predicting the risk of recurrence and prognostic survival of patients more accurately and effectively, and also providing a solid foundation for subsequent clinical decision-making by physicians.

162 patients were split into training and validation groups in this study at a 7:3 ratio. 11 clinically common and easily available variables, including CA199/GGT, AJCC 8th edition TNM staging, Lymph node invasion, Differentiation, Tumor size, CA199, Gender, GPR, PLR, Drinking history, and Complications, were screened using Lasso regression in the training group. The screened risk variables were used to create the Lasso-Cox and RSF models. Additionally, the VIMP approach was used to rank the importance of 11 variables in the RSF model, and the RSF model’s predictive capability was assessed. The prediction capabilities of the two models were compared to choose the most optimal one. In comparison to the Lasso-Cox model, the RSF model has a lower C-index (0.719 vs. 0.845). At the same time, the Lasso -Cox regression model is the optimal model (*P* = 0.019) according to the Delong test. Furthermore, Lasso regression outperforms univariate analysis in terms of addressing the issue of multicollinearity among variables. Therefore, the Lasso-Cox regression model was determined to be the best option. To assess the discriminative and accuracy performance of the model, tdROC, calibration curves, and DCA were performed. Subsequently, the validation group incorporated the Lasso -Cox model for validation. The validation group performed a second calculation of the C-index for the two models, and the results revealed that the Lasso-Cox model (C-index = 0.867) had a higher C-index than the RSF model (C-index = 0.762) and that the predicted results of the calibration curves for 1 year, 3 years, and 5 years were reasonably consistent with the actual results. The 1-year, 3-year, and 5-year tdAUC of the prediction models were 0.841, 0.871, and 0.901, respectively. Greater net returns were generated using the Lasso-Cox regression model in DCA over a wider range of threshold probabilities. Based on the Lasso-Cox model and the variable scores, a visual nomogram was constructed. The three straight lines denoting the anticipated probability of recurrence were crossed by a vertical line at the site of the appropriate total score. The expected 1-, 3-, and 5-year RFS values are represented at the junction points.

It is recognized that a poor prognosis in AC is related to drinking history, complications, tumor size, and CA19-9 [26–28]; The important criteria for assessing tumor progression and guiding patient follow-up is currently AJCC 8th edition TNM staging, lymph node invasion, and differentiation, which was currently uncontroversial and doesn’t need more explanation [28, 29].

The prognostic value of inflammatory parameters in cancer is somewhat controversial, and the mechanisms are still unclear. The following inflammatory markers are now known to be associated with digestive malignancies and have been demonstrated in the literature: CA19-9/GGT [14], PLR [15], NLR [16], AAPR [17], GLR [18], GPR [19]and AGR [20]. Inflammatory markers and prognosis survival in AC are correlated, according to an important amount of research on the subject [30].

Firstly, the most conventional host-tumor interaction in cancer patients is the systemic inflammatory response [31, 32]. All phases of tumor growth, including start, progression, malignant transformation, metastasis, and treatment resistance are all influenced by the inflammatory response [33]. On the one hand, the majority of malignant tumors cause a transcriptional program to attract leukocytes, produce pro-oncogenic chemokines and cytokines, stimulate angiogenesis, and decrease albumin synthesis in the liver, which results in an intrinsic inflammatory response. On the other hand, the tumor microenvironment increases the levels of pro-inflammatory mediators and signaling molecules in the signaling pathways that drive angiogenesis and support tumor activity [30]. As a result, there are more neutrophils and platelets in the blood, whereas there are fewer lymphocytes and higher amounts of albumin. As a result, a poor prognosis is slightly related to elevated inflammatory loads in cancer patients [34].

There are several potential limitations in this study. The first limitation of this study was that it was a single-center retrospective analysis using data from the same hospital and only including Chinese patients. However, differences in the level of care, clinical practice, and patient management styles among different healthcare institutions, as well as the influence of treatment modalities and preferences specific to this study center, may result in a limited representative sample. The results may not be generalizable to other countries or populations. Furthermore, this dataset contains a small sample size of surgically treated AC patients, which affects the generalization of the results as well as the external validity of the study. As a result, several multicenter datasets with high sample sizes are required for future validation.

A nomogram based on the Lasso-Cox regression model was constructed in this study to evaluate patient risk for postoperative recurrence. Compared to models created using other methods, the Lasso-Cox regression model is more reliable and accurate. Additionally, the construction of this nomogram has a unique reference value for physicians to visualize and analyze each patient’s recurrence risk and make timely and accurate clinical decisions, which is extremely important for the identification of patients with a high risk of recurrence of AC and subsequent treatment.

### Electronic supplementary material

Below is the link to the electronic supplementary material.

Supplementary Material 1

## Data Availability

The datasets used and/or analyzed during the current study are available from the corresponding author upon reasonable request.
