# Supplementary material for: Predicting the unpredictable: a robust nomogram for predicting recurrence in patients with ampullary carcinoma
Source: BMC Cancer. 2024 Feb 15;24:212. doi: 10.1186/s12885-024-11960-0 (PMC10870520; doi:10.1186/s12885-024-11960-0)
Supplement: Supplementary file 1 — Supplementary Material 1 [file 12885_2024_11960_MOESM1_ESM.docx]

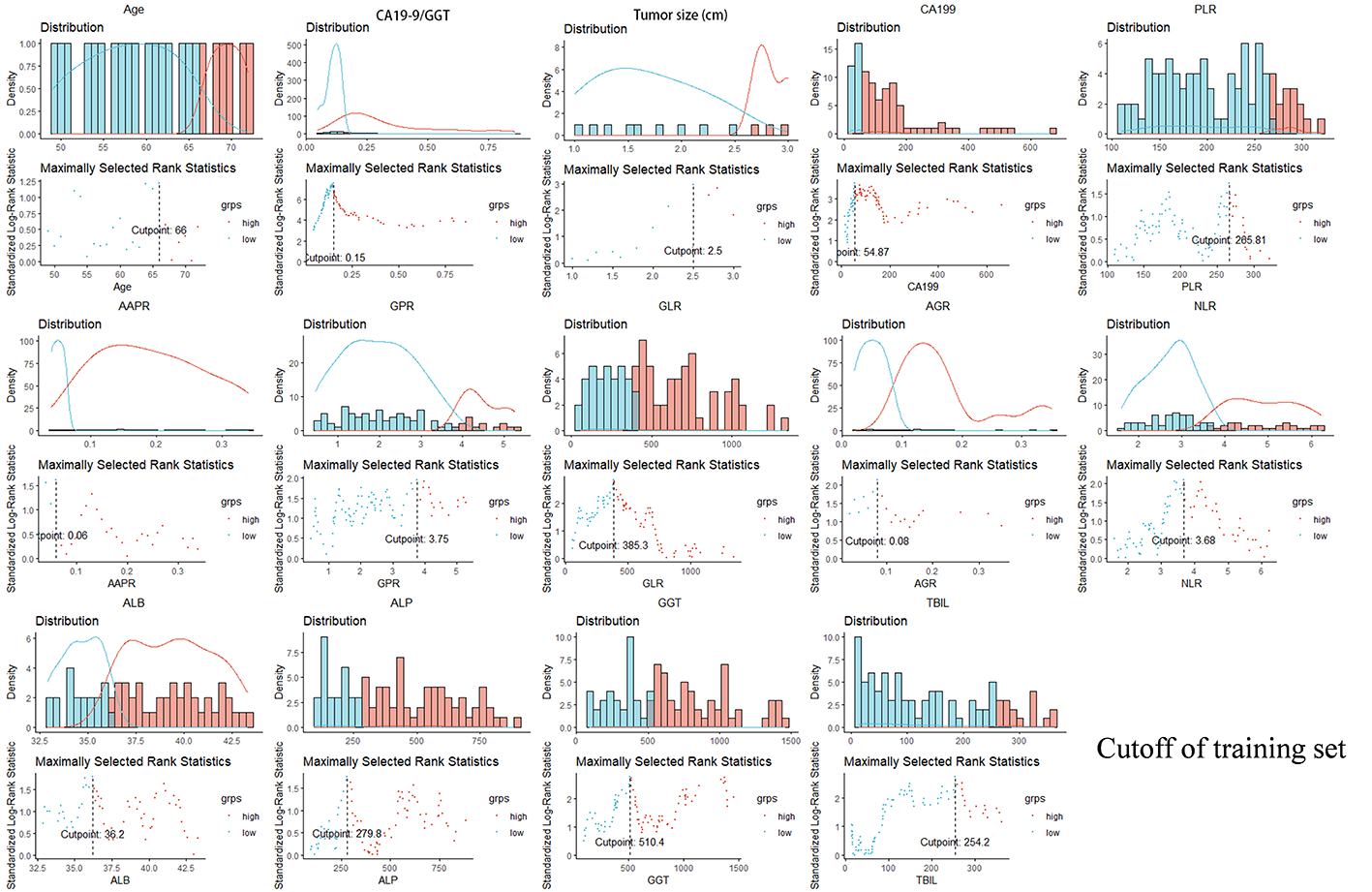


**Supplementary Figure 1:** Cutoff of training set


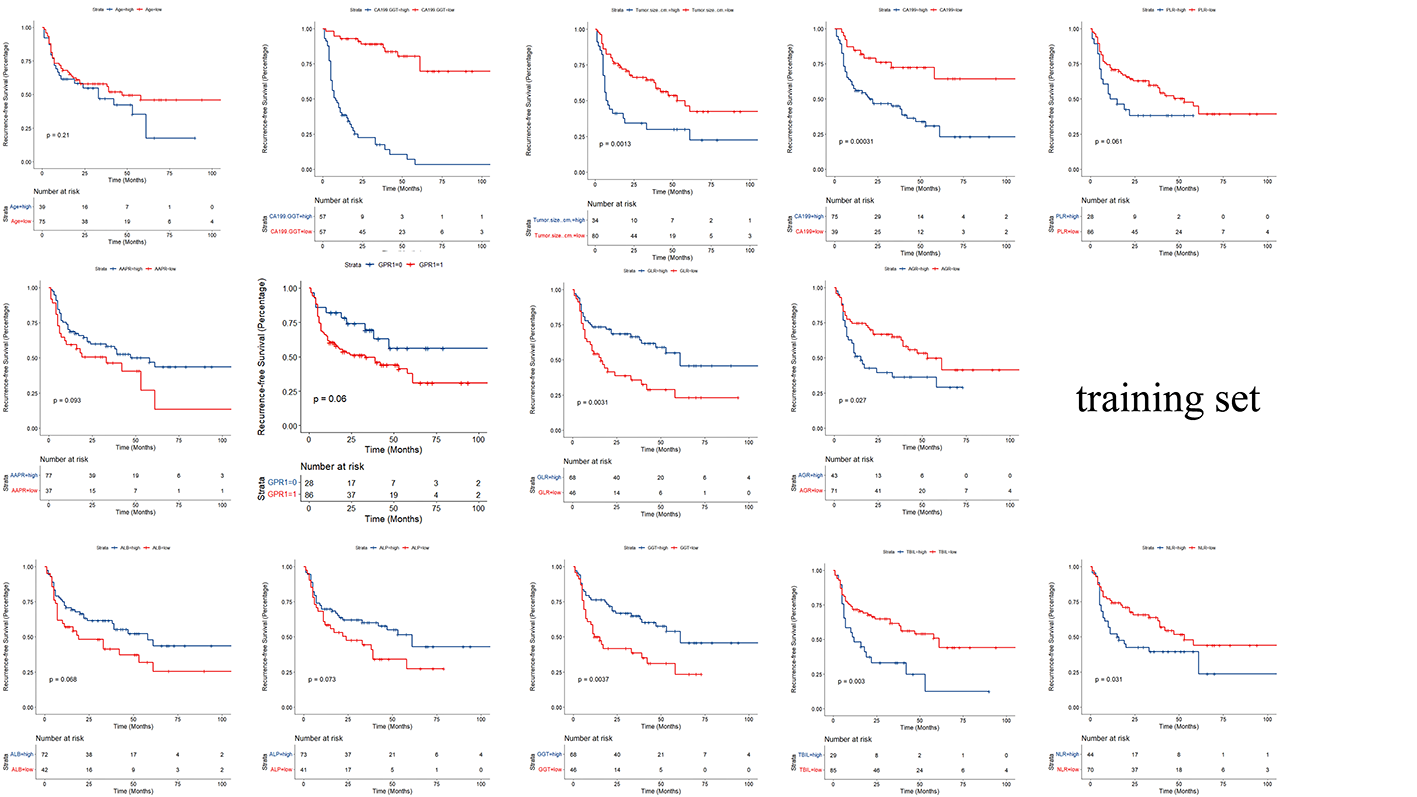


**Supplementary Figure 2:** K-M survival curve of training set


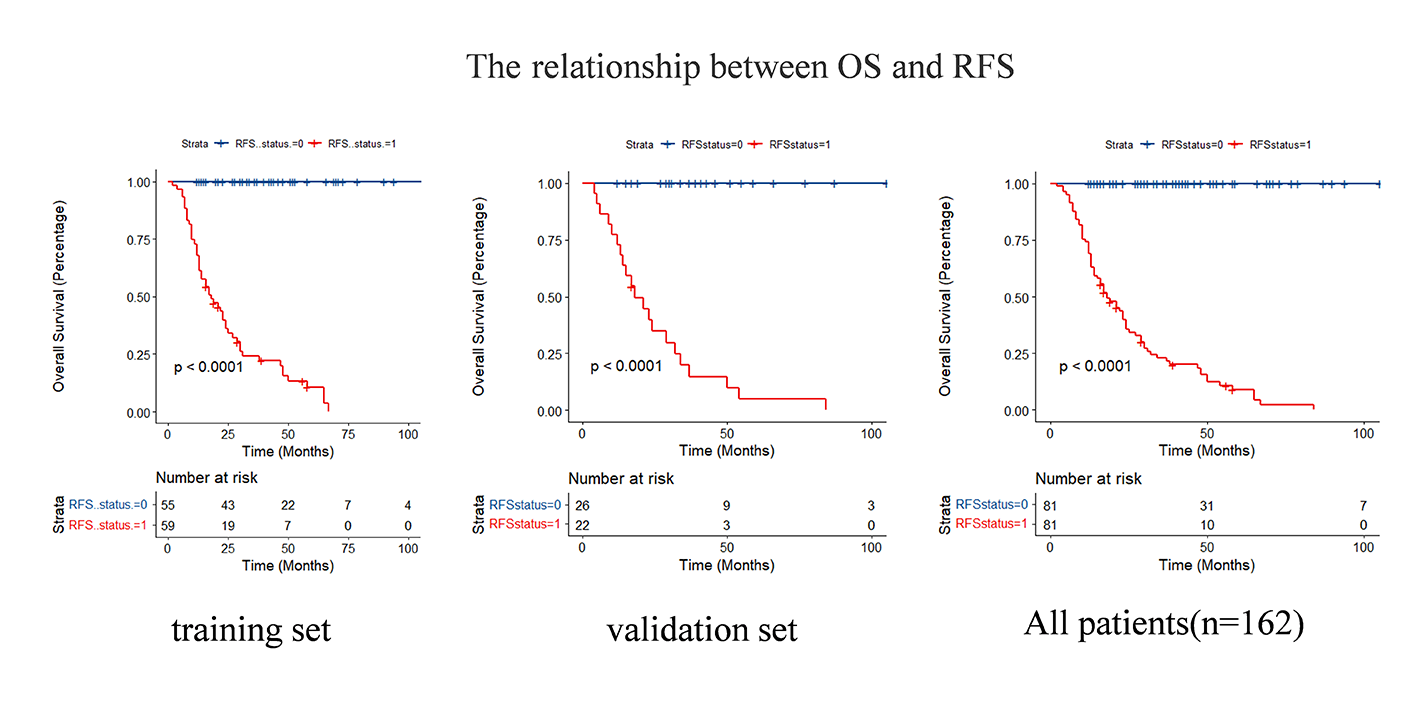


**Supplementary Figure 3:** The relationship between OS and RFS;

**Note:** 0：not recurrence; 1: recurrence


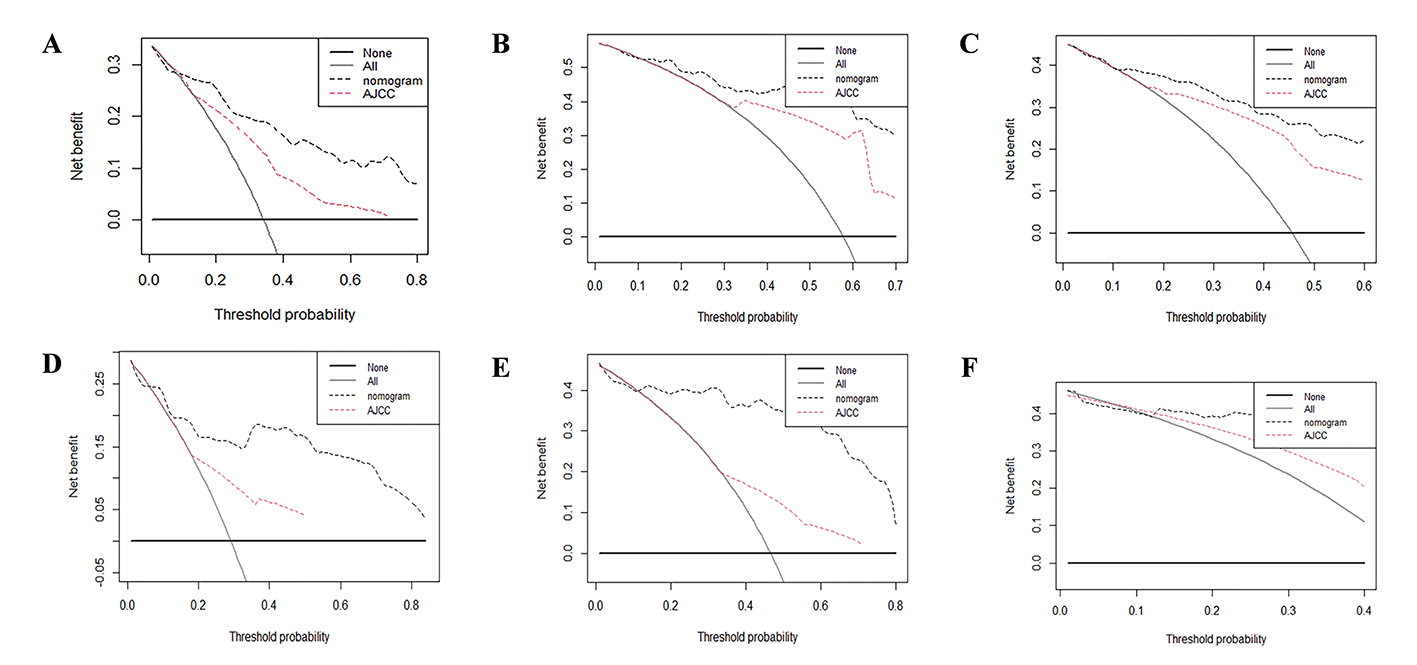


**Supplementary Figure 4:** Decision curve analysis plots of evaluated the prediction performance of the 1-, 3-, and 5-year RFS of Lasso-Cox regression model in the training set and validation set. (A–C) training set; (D–F) validation set. RFS, recurrence-free survival.


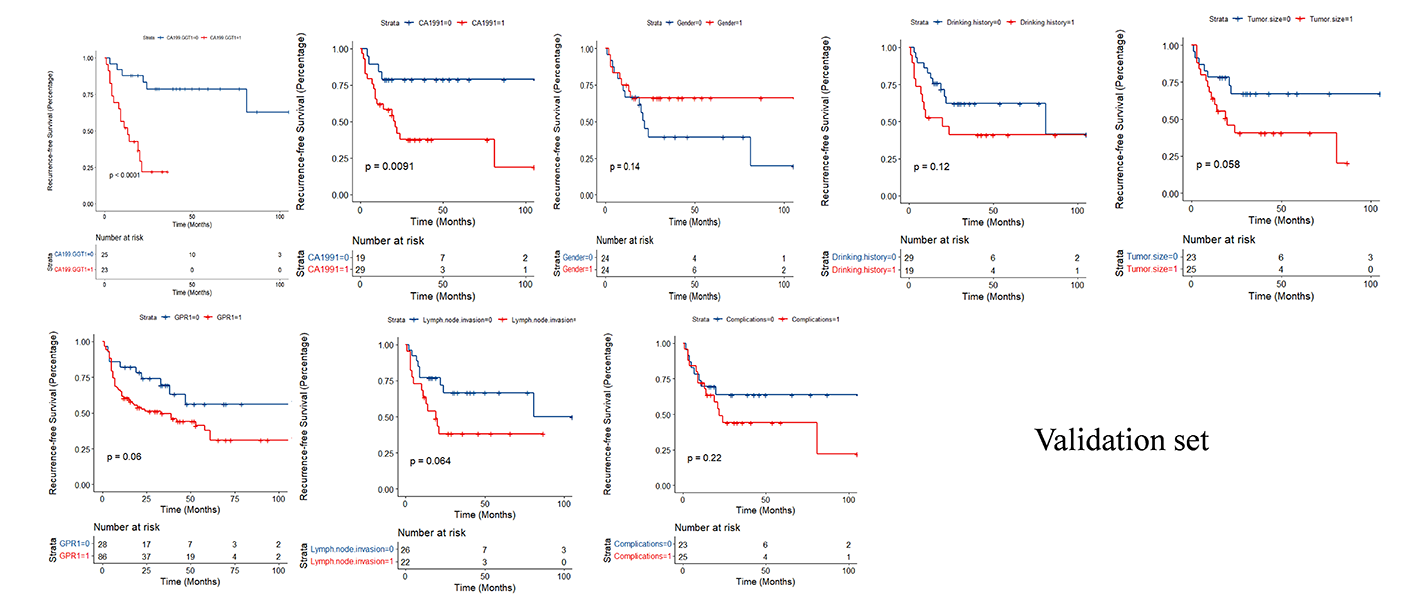


**Supplementary Figure 5:** K-M survival curve of validation set;

**Note:** 0: factor≤cutoff ; 1: factor＞cutoff


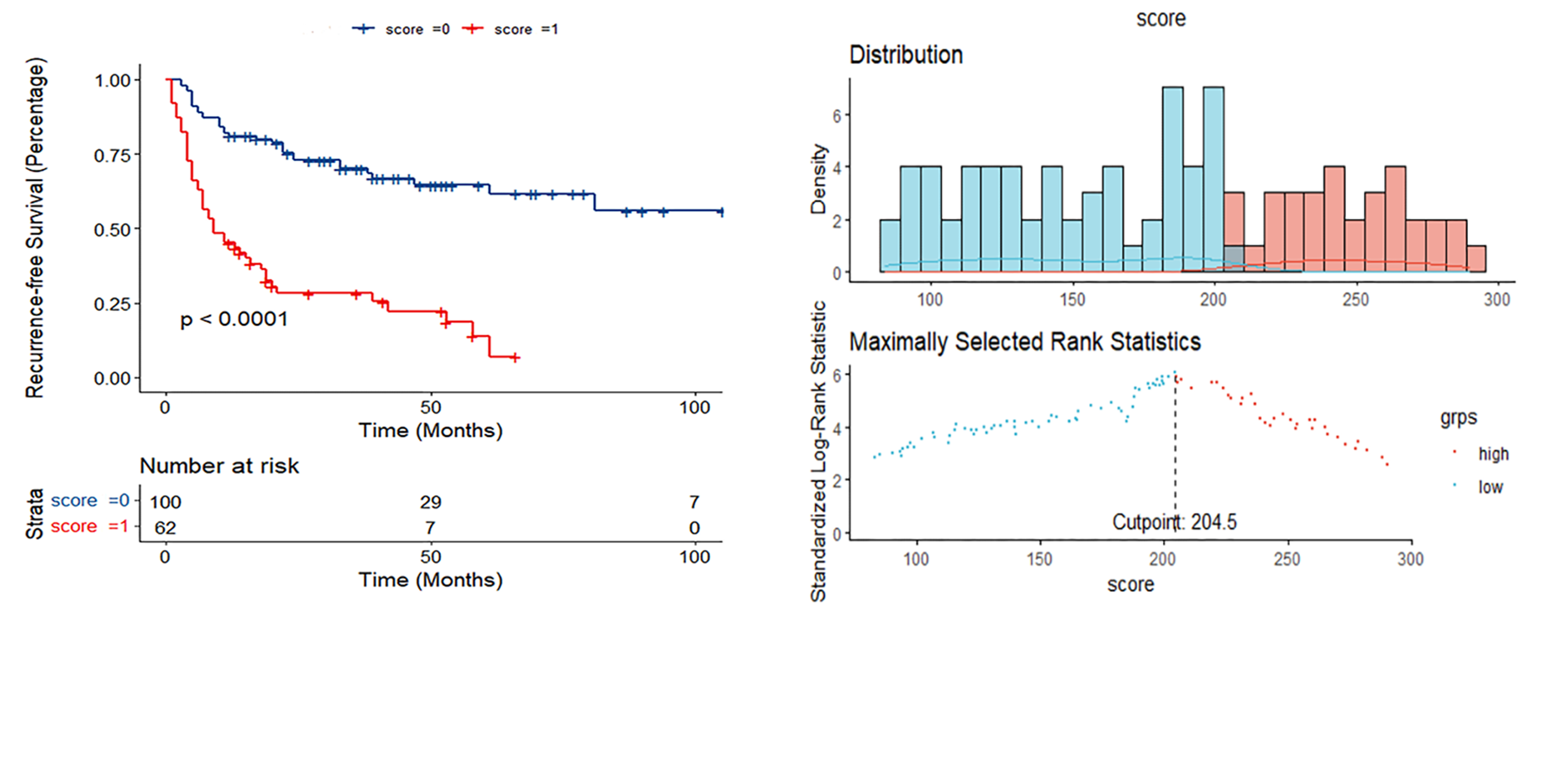
 **Supplementary Figure 6:** K-M survival curve of evaluated the prediction performance of RFS based on Lasso-Cox regression model; The optimal cut-off of **total points** is 204.5.
